# Gold Nanoparticle Monolayers from Sequential Interfacial Ligand Exchange and Migration in a Three-Phase System

**DOI:** 10.1038/srep35339

**Published:** 2016-10-20

**Authors:** Guang Yang, Daniel T. Hallinan

**Affiliations:** 1 Florida State University, Aero-Propulsion, Mechatronics & Energy Center, 2003 Levy Avenue, Tallahassee, FL 32310, USA; 2 Florida A&M University-Florida State University College of Engineering, Department of Chemical and Biomedical Engineering, 2525 Pottsdamer Street, Tallahassee, FL 32310, USA

## Abstract

Using a three-phase system, centimeter-scale monolayer gold nanoparticle (Au NP) films have been prepared that have long-range order and hydrophobic ligands. The system contains an interface between an aqueous phase containing Au NPs and an oil phase containing one of various types of amine ligands, and a water/air interface. As the Au NPs diffuse to the water/oil interface, ligand exchange takes place which temporarily traps them at the water/oil interface. The ligand-exchanged particles then spontaneously migrate to the air/water interface, where they self-assemble, forming a monolayer under certain conditions. The spontaneous formation of the NP film at the air/water interface was due to the minimization of the system Helmholtz free energy. However, the extent of surface functionalization was dictated by kinetics. This decouples interfacial ligand exchange from interfacial self-assembly, while maintaining the simplicity of a single system. The interparticle center-to-center distance was dictated by the amine ligand length. The Au NP monolayers exhibit tunable surface plasma resonance and excellent spatial homogeneity, which is useful for surface-enhanced Raman scattering. The “air/water/oil” self-assembly method developed here not only benefits the fundamental understanding of NP ligand conformations, but is also applicable to the manufacture of plasmonic nanoparticle devices with precisely designed optical properties.

Self-assemblies of noble metal nanoparticles (NPs) display unique chemical and physical properties that are considerably different from either single nanoparticles, or their bulk material counterparts^1^,^2^. Therefore, they have been considered as a new category of functional materials^3^ and readily found a broad range of applications, such as electronics, optics, catalysis and sensors^1^,^4^,^5^,^6^,^7^,^8^. The novel properties of this class of materials arise not only from quantum confinement in discrete NPs, but also from interparticle coupling^9^,^10^,^11^. Thus, in order to fully employ their novel collective properties, precise control over the NP size, shape, and interparticle space is required^1^,^5^.

In the past decade, significant efforts have been dedicated to NP synthesis^12^. Particularly, different colloidal synthetic routes have been developed, leading to an accurate control over NP size^13^, shape^14^, and surface chemistry^15^. It still remains a major challenge to flexibly tune the relative NP position over a macroscopically large area within the assembled functional structures^9^,^16^. Numerous approaches have been taken to control interparticle distance in NP assemblies, of which drop casting is the simplest. For example, Pileni, *et al*. fabricated monolayer silver sulfide NP structures by evaporating a drop of alkylthiol passivated NPs in hexane solution on a solid wafer^17^. It was found that the separation between adjacent NP centers could be tuned by NP size and alkyl chain length. Despite its simplicity, this approach lacks the ability to assemble NPs into large area, thus limiting its capability in large scale device fabrication^18^,^19^,^20^. On the other hand, the Langmuir-Blodgett (LB) technique allows large-scale, two-dimensional (2D) NP monolayers to be transferred from an interface^19^. One requirement of this strategy is to float hydrophobic ligand-capped NPs at the air/water interface to form monolayer NP films^21^. A limited number of methods exist to synthesize hydrophobic metallic NPs^12^,^22^.

Recently, the planar interface of two immiscible liquids (e.g., water and oil) has been found ideal to assemble aqueous NPs into large-area, 2D films^16^,^20^,^21^,^23^,^24^,^25^,^26^. Vanmaekelbergh and others found that aqueous gold nanoparticles (Au NPs) could spontaneously form single layer films at a heptane/water interface by addition of ethanol as an inducer^25^. Although this approach has been found possible to form large area monolayer NP films in a few minutes^24^,^25^, inhomogeneous microscopic features and a large number of voids were often observed in the resulting films^21^. This disordering is possibly because of the broad NP size distribution (e.g. 13 ± 1.5 nm) and long-range, particle-particle repulsion due to residual electrostatic charge on the surface of the particles^21^,^25^. Later, Park *et al*. increased the localized ordering of the Au NP film formed at a hexane/water interface by introducing long-chain alkylthiol ligands to the hexane phase. Once trapped at the interfacial hexane/water region, the Au NPs were *in situ* passivated by the alkylthiol ligands, which decrease the Au NP surface charge and hence the electrostatic repulsion. Despite the enhanced Au NP film ordering using this approach, the monolayer Au NP films usually deteriorate into random islands structure upon the evaporation of the volatile hexane layer^20^,^26^. In addition, successful assembly of monolayer films is extremely sensitive to the ligand concentration in the organic phase, such that Au NP monolayers have low ligand loading. Higher loading can be achieved with the two-phase technique by increasing the ligand concentration in the organic phase, but this leads to multilayer films rather than monolayers. In this work, we present a method to, for the most part, decouple monolayer formation from ligand concentration. This is achieved by performing the self-assembly at a three phase interface. Ligand exchange occurs at a liquid-liquid interface followed by spontaneous translocation of the functionalized nanoparticles to a liquid-air interface where monolayer formation occurs. This approach is also not hindered by evaporation of the organic phase, which is a concern in two-phase assembly approaches^16^,^27^.

New approaches are being actively explored to meet the needs of assembling nanoparticles into functional structures with long-range order and controllable collective properties. Previously, we proposed a “drain-to-deposit” strategy to transfer monolayer Au NP films formed at a hexane/water interface onto solid wafers while maintaining the film integrity^20^. The interparticle space could be flexibly tuned through introducing primary alkylamine ligands of different lengths to the hexane phase. In previous publications we discuss the advantages of using amine ligands, which have similar stability to thiol ligands but a more predictable gold bond that allows for subsequent functionalization^20^,^28^. The current study demonstrates an easy but efficient method of fabricating large area (up to cm^2^ scale) monolayer Au NP films with adjustable interparticle center-to-center distance in an air/water/oil three-phase system. Here we define the “oil” as a type of organic solution that is immiscible with water. Similar to our previous study, we found that aqueous Au NPs spontaneously segregate to the water/oil interface where surface functionalization with amine ligand occurs, preventing the particles from re-entering the aqueous phase. The surface-functionalized Au NPs spontaneously migrate from the water/oil interface to the water/air interface. At the water/air interface, additional rearrangement occurs resulting in an ordered centimeter-scale film. The beauty of this technique is that the ligand exchange reaction and superlattice self-assembly are largely decoupled due to their occurring at separate interfaces. However, the technique does not require additional synthetic steps. In fact, the progression from one interface to the next is driven thermodynamically by the minimization of the system Helmholtz free energy. Unlike our previous publication^27^, the monolayer structures that form are much less sensitive to the ligand concentration in the oil phase. This is due to the fact that the extent of ligand exchange is controlled by kinetics (the particles appear to reside at the water/oil interface for a mere fraction of a second), whereas monolayer formation at the water/air interface achieves equilibrium. This decoupling of interfacial ligand exchange from monolayer formation is a significant advancement in the production of monolayer NP synthesis.

The monolayers assembled with this three-phase technique were characterized and a possible application explored. The interparticle center-to-center distance was tuned by incorporating amine ligands of various lengths in the oil phase. The local ordering and the global ordering of the Au NP films were examined by electron microscopy and X-ray scattering, respectively. Due to their well-controlled ordering, these monolayer Au NP films exhibited interparticle space dependent-surface plasmon resonance (SPR). Finite-difference time domain (FDTD) simulation indicates that the electromagnetic field was greatly enhanced (“hotspots” were generated) between adjacent Au NPs when the film is at resonance with the incident wave. Due to the well-arranged hotspots, the monolayer Au NP films in this study showed spatially consistent surface-enhanced Raman scattering (SERS) across a large area. These findings are promising in opening up a new avenue to fabricate photonic and plasmonic devices based on NP superlattices in a scaled-up manner.

## Results and Discussion

**Figure 1:**
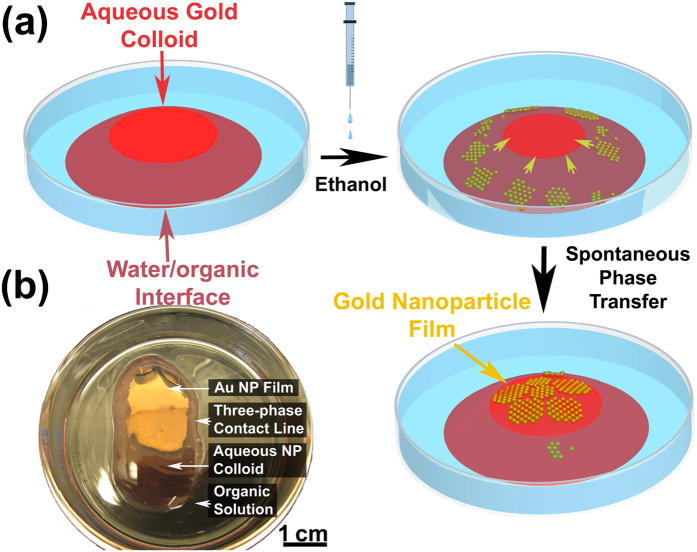
Schematic of the “air/water/oil” three-phase self-assembly strategy to generate Au NP monolayers. (**a**) Clockwise from left: aqueous Au NP colloid was injected to the bottom of a petri dish filled with amine-organic solution. The Au NP colloidal drop protruded into the air so that an “air/water/organic” three-phase system was created. Adding ethanol dropwise to the water/organic interface caused Au NP island structure to form and spontaneously phase-transfer from the water/organic interface to the water/air interface (dark yellow arrows indicate Au NP assemblies moving directions), to form a large area Au NP film at water/air interface. (**b**) Optical photograph of the resulting Au NP film.

Figure 1(a) schematically illustrates the formation of Au NP films in an air/water/oil three-phase system. Initially, aqueous Au NP colloid was electrostatically stabilized by negative citrate ions during synthesis^29^. The addition of ethanol destabilizes aqueous Au NPs and drives them to the water/oil interface, where alkyl ligands attach to the Au NP surface displacing citrate ions. It has been shown that the Au NPs at such an interface have a Janus structure, the top of which (facing the oil phase) is passivated by alky ligands, whereas the bottom face retains residual citrate ions^26^,^28^. The Au NPs appear to form island structures (Fig. 1(a) upper right) as they rapidly migrate from the water/oil interface to the water/air interface (Video S1). At the water/air interface, the Au NPs spontaneously rearrange to eliminate free volume along the film boundary. The whole process takes less than 10 minutes. Consequently, an Au NP film of a large area was gradually generated at the water/air interface (Fig. 1(a) lower right). The Au NP film fabricated in this way, as shown in Fig. 1(b), occupies a large area (cm^2^). Afterwards, the Au NP films were deposited on solid wafers using the method previously reported^20^. The resultant Au NP monolayers have a gold sheen appearance in reflectance, whereas when viewed in transmittance, those films were blue. This is in agreement with similar studies^21^,^30^.

**Figure 2:**
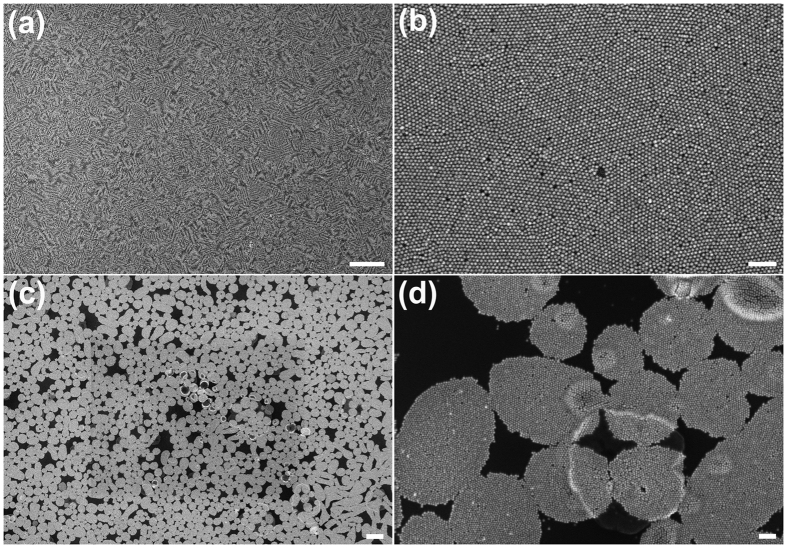
(**a**) SEM image of the monolayer C18-Au NP film assembled in air/water/oil three-phase system with the chloroform/hexane (1:1 by volume)- amine solution as the oil phase and (**b**) SEM image of larger magnification of (**a**). (**c**) C18-Au NP monolayer formed in the three-phase system, with the chloroform-amine solution as the oil phase and (**d**) the magnified SEM image of (**c**). The scale bar is 1 μm for (**a,c**), and 100 nm for (**b,d**), respectively.

The long-range order of the Au NP films was also characterized using SEM. Figure 2(a,b) show typical SEM images of part of the monolayer Au NP film deposited on a silicon wafer. As shown in Fig. 2(a), the SEM image at low magnification (e.g. 10,000×), without resolving individual Au NPs, exhibits moiré interference patterns, indicating the Au NP film has long range HCP “crystal” order^31^. We propose that these moiré patterns are due to interference between the incident electron beam and that reflecting off the silicon substrate, much like an optical moiré pattern can be formed by a single pattern held near a mirror. Various moiré fringe orientations can be observed due to different grain orientations of the hexagonal superlattice with respect to the vector of the incident electron beam. The local Au NP HCP superlattice is further confirmed by SEM images of larger magnifications (Fig. 2(b)). This image was taken from the central area of the moiré interference pattern in Fig. 2(a). It is clear from Fig. 2(b) that few voids exist on the Au NP film. The imperfections that are present are attributed to the finite size distribution of the Au NPs and to residual charge on the Au NPs^20^,^28^.

**Figure 3:**
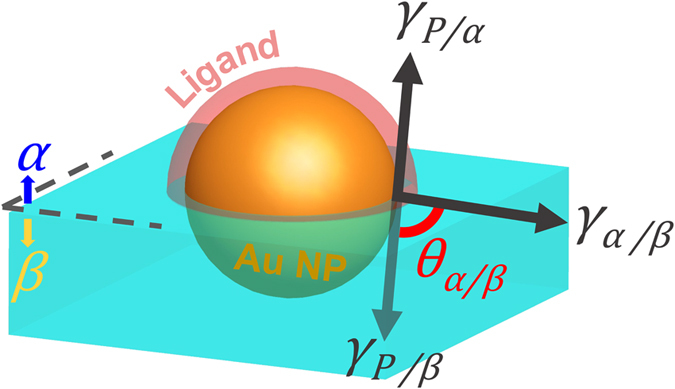
Schematic illustration of a spherical nanoparticle at an interface formed by Phase α and Phase β. The contributions to the changes in the interfacial energy density by placing a particle at the α/β interface include the particle/α interfacial energy density (γ_P/α_), the particle/β interfacial energy density (γ_P/β_), and the α/β interfacial energy density (γ_α/β_). The contact angle, θ_α/β_ is measured in the β phase.

Why did the Au NP film simultaneously translocate from the oil/water interface to the water/air interface? In a two-phase system, the driving forces for the formation of nanoparticle films at interfaces have been intensively studied^16^,^25^,^32^,^33^,^34^,^35^,^36^. It has been commonly accepted that the major driving force for the attachment of the nanoparticle to the liquid/liquid interface or liquid/air interface is the minimization of the Helmholtz free energy^33^,^34^,^35^,^36^. In order to explore the driving force for the Au NPs to translocate from the water/oil interface to the water/air interface, we focus on a single Au NP at the interface between two phases. Shown in Fig. 3, we define the water phase as Phase *β*, the oil or air phase as Phase *α*. According to Pieranski, the change in the interfacial energy due to the translocation of a single nanoparticle from the aqueous phase to the oil/water interface can be written as^37^ (also see Supporting Information)


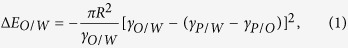


where *γ*_*P/W*_ is the interfacial energy density at particle/water interface, and *γ*_*P/O*_ is the interfacial energy density at the particle/oil interface. Likewise, the interfacial energy density change due the placement of a single particle at water/air (from the aqueous phase) is given by


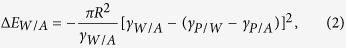


where *γ*_*P/W*_ is the surface tension at particle/water interface, and *γ*_*P/A*_ is the surface tension at the particle/air interface. Based on Young’s equation, the relationship between the contact angle, *θ*_*α/β*_ and the three contributions to the changes in the interfacial energy density can be written as^38^


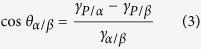


It has been shown that the highest attachment energy of the particle to the *α/β* interface occurs when the particle wets both phases equally, i.e. *θ*_*α/β*_ = 90°^32^,^35^. In fact, wetting experiments confirmed that the contact angle of approximately 90° is required to form an Au NP film at *α/β* interface^24^,^25^. For example, Binks *et al*. calculated the partition profile of 10 nm particles in water/oil biphasic system, and showed that if the contact angle slightly deviated from 90°, the nanoparticles may be pulled to bulk phase. For angles <90°, the particles easily enters Phase *α* and for *θ*_*α/β*_ >90° the reverse is true^21^,^35^. Thus, it is reasonable to assume a contact angle of 90° for Phase β being oil in this study. Substituting *θ*_*α/β*_ = 90° into Equation (3), the following relationship is obtained


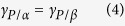


Substituting Equation (4) into Equation (1) yields


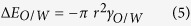


Since even if the contact angle *θ*_*α/β*_ ≤ 90° it is still not possible for Au NPs to enter air phase (*α* is air). Thus, it is controversial to directly apply Equation (4) for an air *α* phase. Rearranging Equation (2) yields


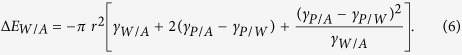


The Au NPs are capable of staying at the water/air interface, only if the contact angle, *θ*_*W/A*_ ≤ 90°. From Equation (3), it is easily seen that *γ*_*P/A*_* ≥ γ*_*P/W*_. Substituting it into Equation (6) yields


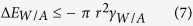


At room temperature, the water/air surface tension *γ*_*W/A*_ = 71.9 mJ∙m^−2 ^39 is 21.5 mJ∙m^−2^ larger than that of oil/water at *γ*_*O/W*_ = 50.7 mJ∙m^−2^ for the hexane/chloroform mixture (1:1 by volume, Table S1). Based on Equations (5) and (7) for particles of the same size, *ΔE*_*O/W*_ > *ΔE*_*W/A*,_ i.e. the particle at the water/air interface has a lower Helmholtz free energy than at the oil/water interface. The difference between these two Helmholtz free energies offers the driving force for nanoparticles transferring from the oil/water interface to the water/air interface.

The analysis above indicates that an aqueous nanoparticle, once trapped in oil/water interface, will spontaneously translocate to the water/air interface, as long as the condition *ΔE*_*O/W*_ > *ΔE*_*W/A*_ is satisfied, regardless of the organic solvent type, nanoparticle type and size. To test this hypothesis, different organic solvents were employed to form the air/water/oil three-phase system with the same aqueous Au NP colloid. The organic solvents have a smaller oil/water interfacial energy than that of the air/water (Table S1). Octadecylamine (denoted C18-NH_2_) was used as the capping ligand. In all cases, a monolayer C18-Au NP film could be formed at the air/water interface using the three-phase assembly method (Figure S1), verifying the hypothesis. It is also worth emphasizing that the aqueous Au NPs of different sizes are capable of forming the monolayer Au NP films using the three-phase assembly method based on the statement of *ΔE*_*O/W*_ > *ΔE*_*W/A*_. The results will be discussed in a future publication.

It should be emphasized here that in order to achieve an optimistic ordering in a monolayer Au NP film, the organic solution (i.e. oil phase) should be carefully selected. An organic mixture of chloroform and hexane (1:1 volume ratio) was found most suitable for assembling ordered monolayer Au NP films. Irregular long-range structures were formed with solvents whose surface tension and volatility were not tuned to produce long-range order. As shown in Fig. 2(c), a local pattern of oval arrays was created if a chloroform/C18-NH_2_ solution (10.4 μM) was used. The SEM image of higher magnification indicates that the oval was composed of hexagonally packed Au NPs, as shown in Fig. 2(d). The variation of the grain size of the Au NP film with the type of oil phase can be attributed to a matching of the oil and water dispersion force. More specifically, the expression of any surface tension, *γ*, (versus air) can be expressed as the sum of components resulting from dispersion forces, *γ*^*d*^, and polar forces, *γ*^*p*^ 40.


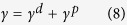


The interfacial tension between water and oil is then expressed in terms of these two components for each phase


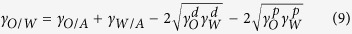


where *γ*_*O/W*_, *γ*_*O/A*_ and *γ*_*W/A*_ are the surface tensions at oil/water interface, oil/air interface and water/air interface, respectively; *γ*_*O*_^*d*^ and *γ*_*W*_^*d*^ are the dispersion components in the surface tensions of oil and water; and *γ*_*O*_^*p*^ and *γ*_*W*_^*p*^ are the polar components in the surface tensions of oil and water, respectively. The calculated interfacial tension of chloroform/water is 50.7 mJ∙m^−2^ (Supporting Information Table S1), almost twice as much as that of chloroform/air at 26.7 mJ∙m^−2^, both of which are smaller than that of water/air at 71.9 mJ∙m^−2^ (at room temperature)^39^,^41^. If the sum of the oil/water and oil/air surface tensions is less than the water/air surface tension (*γ*_*O/A*_ + *γ*_*O/W*_ < *γ*_*W/A*_), then oil will wet the surface of the water. Of all the organic solvents used in this study, hexane is the only oil to satisfy this condition (see Table S1). As shown in Figure S1(a), the hexane then evaporates from the water surface inducing instabilities in the Au NP superlattice structure and causing large voids in the monolayer. On the other hand, without any oil wetting the surface of the water, the Au NPs appear not to acquire sufficient mobility for complete rearrangement into a monolayer with long-range order (see Figure S1(b–d)). When *γ*_*O/A*_ + *γ*_*O/W* = *γW/A*_, as is the case for 1:1 hexane:chloroform mixture, the free energy of the water/oil/air interfaces is the same as that of the water/air interface. In this case, both oil mobilized Au NP film and water/air regions can coexist. This allows for rearrangement to an equilibrium HCP structure with long-range order driven by minimization of *lateral* interfacial energy.

The surface tension (*γ*_*O/A*_^*mix*^) of hexane/chloroform mixture can be calculated by an empirical model based on parachor^42^


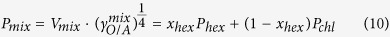


where *P* is the parachor of the hexane/chloroform mixture, *V* is the molar volume of the hexane/chloroform mixture, *x*_*hex*_ is the molar fraction of hexane, *P*_*hex*_ is the parachor of the hexane and *P*_*chl*_ is that of the chloroform, respectively.

The parachor of the pure liquid can be calculated by


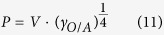


Under room temperature, the molar volumes of hexane and chloroform are 130.6 cm^3^·mol^−1^ and 80.1 cm^3^·mol^−1^, respectively^43^. The surface tension of hexane and chloroform are taken from Table S1. Thus the parachors of hexane and chloroform can be calculated at *P*_*hex*_ = 268.6 cm^3^·mJ^1/4^·mol^−1^ ∙m^1/2^ and *P*_*chl*_ = 182.1 cm^3^·mJ^1/4^·mol^−1^·m^1/2^. For hexane/chloroform mixture (1:1 by volume), *x*_*hex*_ is estimated at 0.38, and the molar volume of the hexane and chloroform mixture is calculated at *V*_*mix*_ = 99.8 cm^3^·mol^−1^, with the density of the hexane and chloroform mixture (1.07 g∙cm^3^) given by ref. 44. Substituting these values into Equation (10), the surface tension of the hexane and chloroform mixture is calculated at *γ*_*O/A*_^*mix*^ = 21.5 mJ∙m^−2^. As reported in Table S1, hexane and chloroform have nearly the same value of *γ*_*O/W*_ at 50.7 mJ∙m^−2^. Thus *γ*_*O/A*_^*mix*^ + *γ*_*O/W*_^*mix*^ = 72.2 mJ∙m^−2^, which is very similar to that of *γ*_*W/A*_ (71.9 mJ∙m^−2^)^39^.

In order to account for the presence of the alkylamine ligand, the simplest case is considered first, in which we assume that the alkylamine is homogeneously distributed in the organic phase. Octadecylamine in hexane/chloroform mixture (1:1 by volume) is used as an example. Based on the molar concentration of amine ligand in the organic phase (10.4 μM), the mole fraction of alkylamine is *x*_*C18*_ = 1.0 × 10^−6^. The parachor of C18-NH_2_ is *P*_*C18*_ = 774.441 cm^3^·mJ^1/4^·mol^−1^·m^1/2 ^45, and that of the organic phase *P*_*org*_ = 215.0 cm^3^·mJ^1/4^·mol^−1^·m^1/2^. Substituting these values into Equation (10), the parachor of the ligand-oil solution is the same as the neat oil within appropriate significant figures. Assuming volume additivity, the molar volume of the ligand-oil mixture is also the same as that of the neat oil, so that the effect of alkylamine ligand on the surface tension of the oil phase is negligible.

The above analysis might be over simplified, because it does not consider preferential partitioning of the alkylamine to the oil/water interface. Therefore, we consider a limiting case, in which we assume that a dense layer of C18-NH_2_ exists at the water/organic interphase so that water is only in contact with C18-NH_2_. Under such circumstances, the relevant surface tension becomes that of ligand/water (*γ*_*C18/W*_) instead of oil/water (*γ*_*O/W*_). The total surface tension of C18-NH_2_ is *γ*_*C18*_ = 30.5 mJ∙m^−2^ according to ref. 46. Since the information of the surface tension components of C18-NH_2_ is lacking, we assume that the dispersion force of C18-NH_2_, *γ*_*C18*_^*d*^, is equal to the surface tension of octadecane at 27.8 mJ∙m^−2 ^47. According to Equation (8), the polar force *γ*_*C18*_^*p*^ = *γ*_*C18*_-*γ*_*C18*_^*d*^ = 2.7 mJ∙m^−2^. Substituting these values into Equation (9), the surface tension of ligand/water, *γ*_*C18/W*_ = 30.2 mJ∙m^−2^. This value is almost half of the oil/water surface tension (*γ*_*O/W*_ = 50.7 mJ∙m^−2^). In this limiting case, the amine surfactant significantly reduces the oil/water surface tension. Thus the driving force (Helmholtz free energy minimization) for Au NPs to migrate from the oil/water to the water/air interface is 20.5 × πr^2^ mJ greater than if the ligand is neglected in the calculation. On the other hand, this limiting case implies that coexistence of hexane + chloroform and Au NPs on the water surface does not occur if the oil/water interface is loaded with ligand. It is still reasonable to expect, even in this limiting case, that Au NPs sequester ligands from the oil/water interface during ligand exchange, providing a source of ligand-free hexane + chloroform to coexist on the water surface.

**Figure 4:**
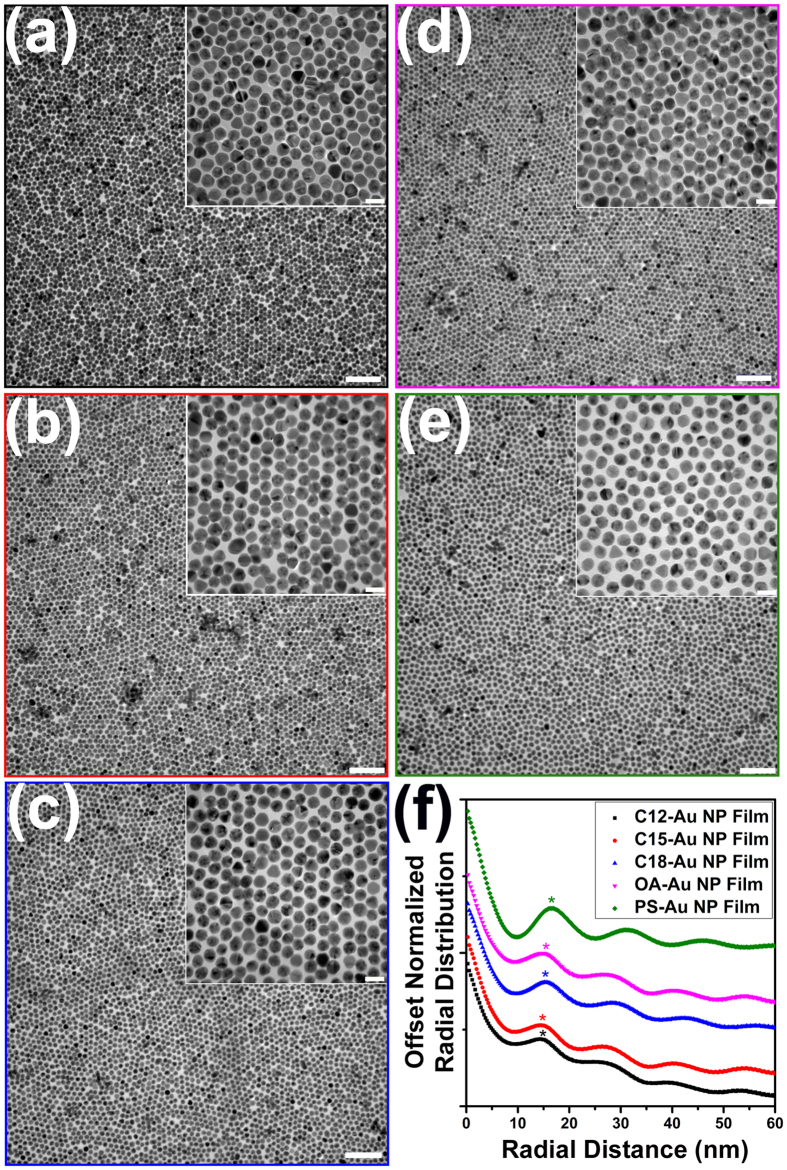
TEM images of monolayer (**a**) C12-Au NP film, (**b**) C15-Au NP film, (**c**) C18 Au NP film, (**d**) OA-Au NP film and (**e**) PS-Au NP film. The insets show magnified images of the corresponding TEM micrographs. The scale bar of the main TEM image is 100 nm and that of the inset is 20 nm. (**f**) The plot of the normalized radial distribution of different Au NP films based on TEM image analysis. The star shows the primary peak of the radial distribution. The curves are offset for clarity.

More detailed studies on the local ordering of the film were performed using TEM. As shown in Fig. 4, all types of Au NP films made by the three-phase self-assembly technique exhibit localized HCP structure. There were no observable cracks across each Au NP film using TEM, manifesting the advantage of using the “drain-to-deposit” strategy to transfer Au NP film from a liquid surface onto a solid wafer^20^,^27^.

The order of the monolayer Au NP films is also affected by the concentration of the amine ligand in the organic phase. In a previous study using two-phase self-assembly, we found that ordered monolayer Au NP films could only be obtained by carefully adjusting the alkylamine concentration in the organic phase^20^. With small increases in ligand length, the alkylamine concentration needed to be decreased by orders of magnitude in order to achieve monolayer Au NP films. A large excess of ligand resulted in multilayer films (Figure S2(c))^20^. The Park group has observed Au NP multilayers formed during self-assembly at a water/oil (two-phase) interface with alkylthiol ligands. Like alkylamine ligands, this occurred at ligand concentrations greater than that optimal for monolayer formation^21^. In contrast, the same ligand concentration has been used for widely varying ligand lengths in this study using three-phase self-assembly. In addition to alkylamines with molecular weight (*M*_*W*_) between 185 and 270 g/mol, this technique has been successfully used with amine-terminated polystyrene (*M*_*n*_ = 5,000 g/mol). The much weaker concentration dependence of this three-phase method is due to the fact that the particle residence time at the water/oil interface, where ligand exchange occurs, is short and immediately followed by migration to the air/water interface, where ligand exchange is suppressed. Employing hydrophobic polymer ligand, such as polystyrene, to tune the interparticle spacing in monolayer films has not, to the best of our knowledge, been reported previously. It is worth emphasizing that even with this three-phase self-assembly method, there exists an upper limit to the ligand concentration above which multilayer Au NP films form. See supporting information for details.

The interparticle center-to-center distance (*d*_*c-c*_) was quantified using pair correlations of TEM images. As shown in Fig. 4(f), the primary peak of the pair correlation indicates the interparticle center-to-center distance for each Au NP film, and the value of d_c-c_ for each Au NP film is summarized in Table 1.

While the TEM images are limited to small regions of the sample, SAXS can be used to probe a large area of the sample. The contribution to the SAXS scattering intensity of each Au NP film can be expressed by^48^


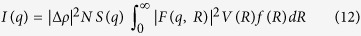


where *I*(*q*) is the intensity scattered to wave vector, *q. Δρ* is the scattering contrast between Au NPs and the surrounding medium; N is the total number of scattering particles; *S(q)* is the structure factor determined by the Au NP monolayer structure. *F*(*q, R*) is the scattering form factor, accounting for scattering due to an individual Au NP; *V(R)* is the particle volume; and *f*(*R*) is the probability of occurrence of a particle of radius *R*.

The structure factor, *S(q)*, can thus be extracted from the raw 1D SAXS scattering data,


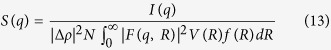


The denominator has been calculated in our previous study for the same Au NPs^28^.

**Figure 5:**
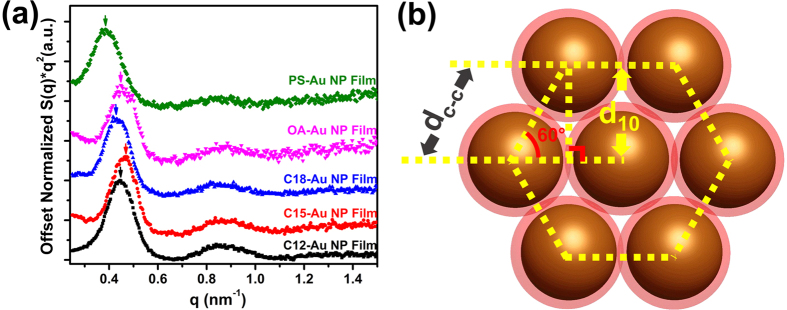
(**a**) 1D plot of *S*(*q*)*q^*2*^ versus scattering vector q of different Au NP films. The data sets are offset for clarity. (**b**) Schematic illustration of the geometric relation (top view) between HCP (10) plane spacing, *d*_*10*_ and the Au NP center-to-center distance, *d*_*c-c*_ in an Au NP film.

As shown in Fig. 5(a), the primary peak of the structure factor, *S(q)*, versus scattering vector, *q,* indicates the q* corresponding to the (10) plane spacing, *d*_10_, in an Au NP film with HCP structure. Based on Bragg’s Law and the geometric relationship (Fig. 5(b)) between the HCP (10) plane spacing (*d*_10_) and the center-to-center distance (*d*_c-c_), *d*_*c-c*_ values of various Au NP films can be obtained^27^. These values are reported in Table 1. It is obvious that the center-to-center distance of all Au NP films measured from TEM images agree decently well with those calculated from 1D SAXS profiles within experimental error. The plot of edge-to-edge distance calculated from d_c-c_ of each Au NP film with theoretical prediction is summarized in the supporting information (Figure S3).

**Figure 6:**
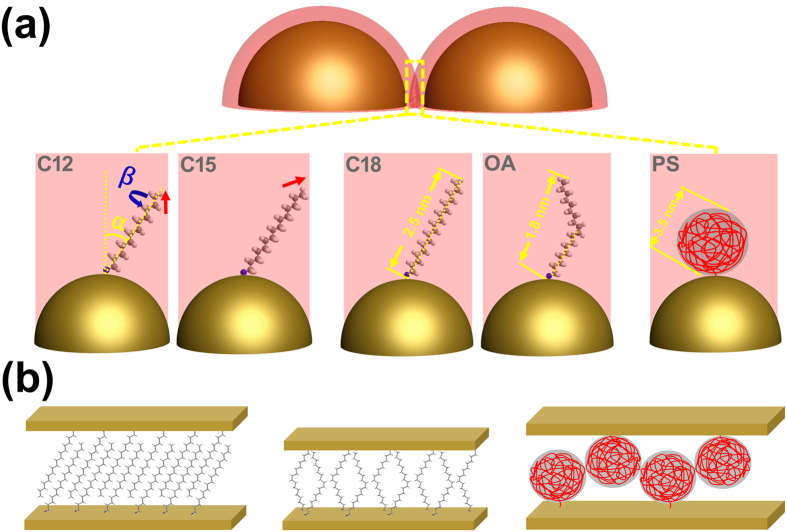
Schematic illustration of the structural configuration of various Au NP surface ligands. (**a**) “Odd-even” effect of the alkylamine ligand on Au NP surface brought by the tilt angle, α and the twist angle, β. The red arrow indicates the direction of the terminal CH_3_-CH_2_- moiety. Different molecular conformations between the octadecylamine (C18-NH_2_) and the oleylamine (OA) on Au NP surface. Amine terminated polystyrene (PS) on Au NP surface with a random coil structure. (**b**) The illustration of the ligand conformation and the relative surface ligand position of two adjacent Au NPs in the NP monolayer for alkylamines (left), oleylamine (middle) and polystyrene (right).

From Table 1, it is interesting to note that the average d_c-c_ decreases a bit from 14.7 ± 0.2 nm for dodecylamine-capped Au NP (C12-Au NP) film to 14.5 ± 0.3 nm for pentadecylamine-capped Au NP (C15-Au NP) film, and increases to 15.3 ± 0.1 nm for C18-Au NP film (based on SAXS analysis). This is different from what we expected based on our previous study, in which the Au NP center-to-center distance, *d*_*c-c*_, increased with the alkylamine chain length^20^. This obvious discrepancy could be possibly explained by the “odd-even” effect^49^,^50^,^51^,^52^. It has been found that the geometry of the self-assembled monolayer (SAM) of alky ligands on a noble metal surface is closely related to the integer number of carbon atoms in the alkyl tail^49^. The geometry of the monolayer alkyl chain can be described by the tilt angle, *α*, and the twist angle, *β*, as shown in Fig. 6(a). The tilt angle (*α*) is formed by the alkyl chain long axis in the plane which includes all the carbon atoms and the Au NP surface normal. The twist angle (*β*) describes the rotation of the alkyl chain long axis. Ramin *et al*.^5^ did a systematic molecular dynamics simulations to predict the structure properties of n-alkanethiols CH_3_(CH_2_)_n-1_SH(C_n_, 4 ≤ n ≤ 22). They found that the tilt angle, *α* is 26.35° ± 0.32° for C12-SH, 3.71° degree smaller than that of the C15-SH. Another contribution comes from the orientation of the terminal CH_3_-CH_2_- moiety of the surface alkyl ligand (Fig. 5(a)). For a monolayer of CH_3_(CH_2_)_n−1_SH (n even), the terminal CH_3_-CH_2_- bond is predominantly perpendicular to the surface, while the terminal methyl groups for alkyl chains of odd carbon atoms is tilted away from the surface normal^49^. Consequently, the latter gives an enhanced out-of-plane rotation of the terminal CH_3_ around its neighboring C-C bond, resulting in a slightly looser packing for the alkyl chain with an odd number of carbon atoms. Thus, the C15 alkyl chains may be able to more completely interdigitate, resulting in a smaller interparticle gap. In our previous report, the C12-Au NP film formed in “water/hexane” interface has a smaller interparticle gap than that of the C15-Au NP film^20^. This difference could be attributed to the different Au NP film fabrication processes and the environments in which the Au NP films were deposited on the wafer and dried, which also contributes to the configuration of tilt angle and the twist angle of the Au NP surface alkyl ligands^51^. It should be noted that since there is relatively sparse studies of “odd-even” effect on the SAM of alkylamines, here we assume that SAM of alkylamine ligands on Au NP surface has a similar alkyl chain configuration with that of the alkylthiolates. This assumption is reasonable, given the fact that alkylamine ligands have a similar manner to the alkylthiol of forming a SAM on Au (111) surface^53^. In fact, the tilt angle of the surface alkyl chains can be dictated by the chain-chain van der Waals interaction and lateral dipole-dipole interaction^54^,^55^. The former has been found strongly dependent on the alkyl chain length^54^, and the latter has been demonstrated similar for both amine and thiol SAMs^55^.

It is also interesting to note that despite having the same number of carbon atoms, the Au NP center-to-center distance of oleylamine-capped Au NPs (denoted OA-Au NPs) is 0.7 ± 0.2 nm smaller than that of the C18-Au NPs. This is because the projection length of a single OA chain onto the Au NP surface normal is shorter than that of a C18-NH_2_ chain due to the existence of the unsaturated carbon-carbon double bond. For OA ligand, the angle between the double bond and its neighboring single carbon bond is 120°. Based on the geometric relation, the length of the projection of OA along Au NP surface normal can be estimated at 1.6 nm, smaller than the projection of C18-NH2 contour to Au NP surface normal (2.2 nm). Here the same tilt angle of 30° was assumed for both C18-NH_2_ and OA^50^.

Another contribution to the difference of the interparticle space between the OA-Au NP film and the C18-Au NP film could be due to diastereoisomerism of OA. We conjecture that the distribution of cis and trans configurations of OA results in a looser packing of ligands. Analogous to that of C15, looser packing allows for more complete interdigitation of OA ligands as compared to C18-NH_2_ ligands. Shown in Fig. 6(b) left panel, the linear C18-NH_2_ chains can pack densely. However, the double bond kink of non-linear OA chains results in less dense chain packing (Fig. 6(d) middle panel). These different ligand configurations inevitably affect the strength of van der Waals interactions between ligands, thus affecting the interparticle space. It is worth noting that the study on the OA ligand scaling behavior based on the interparticle space in the Au NP monolayer in our study agrees very well with the reported value elsewhere (1.5 nm to 1.8 nm)^56^.

The number average molecular weight of the amine terminated polystyrene (PS) is 5000 g/mol, two orders magnitude larger than those of the alkylamine ligands. The contour length of a single PS chain is estimated at *R*_*max*_ = 12.4 nm (refer to Equation S9). Thus, the *d*_*c-c*_ of PS-Au NP film can be estimated at 25.1 nm assuming that the PS ligands are fully interdigitated between adjacent Au NPs^20^. However, this value is 8 nm larger than the measured one (Table 1). This is due to the fact that linear polymer chains in the amorphous state under room temperature have both gauche and trans bond conformations along the backbone^57^. A linear contour configuration is unfavorable in terms of conformational entropy and not appropriate to evaluate the polystyrene chain size. We assume that the whole polystyrene chain is contained in a virtual sphere, as shown in Fig. 6. Based on the ideal polymer equivalent freely joint chain model, the PS virtual sphere diameter, *R*_*F*_, can be calculated from the root-mean-square (RMS) end-to-end distance by^58^


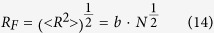


where ***R*** is the polymer chain end-to-end vector, *b* is the Kuhn length and *N* is the number of Kuhn monomers. By definition, *N* is related to *b* by


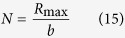


The Kuhn length, *b* can be calculated by


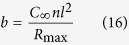


where *C*_*∞*_ is called the characteristic ratio, and is 10.8 at room temperature for polystyrene^59^. *l* is the actual C-C bond length (0.154 nm). *n* is the number of the skeleton carbon bonds, which is calculated by


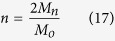


where *M*_*n*_ is the number average molecular weight of polystyrene (5000 g/mol), *M*_*o*_ is the molecular weight of a styrene monomer (104.1 g/mol). Substituting Equation (17) into (16), one obtains the Kuhn length for PS at room temperature as *b* = 1.4 nm. Consequently, the number of Kuhn monomers can be calculated as *N* = 8.9 using Equation (15). Thus, the diameter of the PS virtual sphere on Au NP surface can be estimated at *R*_*F*_ = 4.2 nm. Based on Table 1, the average edge-to-edge distance of the PS-Au NP is 4.3. This suggests that the polystyrene coils on closest neighboring Au NPs interdigitate (Fig. 6(b), right panel). This is the first report that we are aware of directly examining polymer chain scaling using ordered nanoparticles.

**Figure 7:**
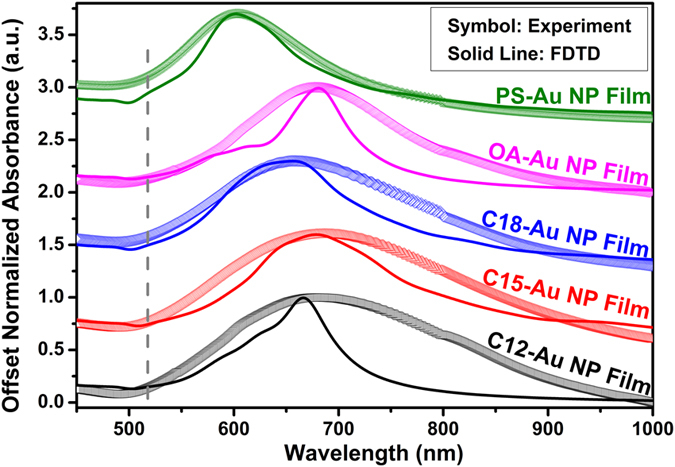
The normalized UV-vis-NIR absorbance of different Au NP films (symbol) and that of the FDTD simulated results (solid line). The vertical dash line indicates the SPR maximum of the aqueous Au NP colloid at 519 nm. Data and simulation curves are offset for clarity.

It is thus seen from the above discussion that ligand length and conformation affects Au NP center-to-center distance, *d*_*c-c*_. Precisely tunable particle spacing is of key importance for developing optical devices composed of nanoparticle assembled structures^60^,^61^. The optical applications of such devices are usually built up based on the near-field interaction of the electromagnetic field (light) and the nanoparticle surface free electron oscillations. When the two are in resonance, nanoparticles exhibit intensive light absorption. This phenomenon is termed localized surface plasmon resonance (LSPR). As has been demonstrated by many studies, the LSPR of nanoparticle assemblies strongly depend on the interparticle spacing^20^,^62^,^63^ and surrounding ligand dielectric constant^20^. Shown in Fig. 7, the SPR maximum for all Au NP films deposited on glass slides experienced significant red-shifts compared with the value of their counterpart dispersed in water (519 nm). This is due both to the sharp decrease of the media dielectric constant (ε_m_) (e.g. (*ε*_*m*_ = 3.07 for C12-NH_2_ and 78.30 for water) and to particle-particle interactions^20^. The SPR maximum also varies with the NP film type. For example, along with the decrease of the Au NP center-to-center distance from 17.0 ± 0.2 nm for PS-Au NP film, to 14.5 ± 0.3 nm for C15-Au NP film, the SPR maximum is red-shifted from 601.7 ± 2.1 nm to 683.6 ± 3.9 nm, indicating a near-field coupling of adjacent Au NPs^20^. The SPR maximum collected from UV-vis-NIR for each Au NP film has been summarized in Table 2. In a previous report, by solely using alkylamine ligand, we were able to tune the SPR between 645 nm (monolayer C18-Au NP film) and 819 nm (monolayer C6-Au NP film)^20^. The Au NP monolayer with amine-terminated polystyrene assembled in air/water/oil three-phase system has an extended SPR down to 601 nm, meaning that we can manipulate the plasmonic response of the Au NP monolayers in an even broader wavelength range than demonstrated previously.

**Figure 8:**
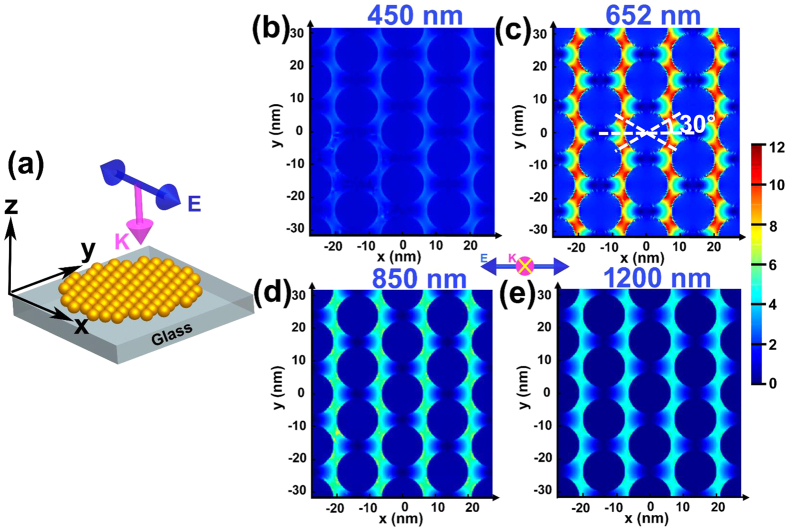
(**a**) Schematic illustration of the model setup for FDTD simulation of the Au NP monolayers. The pink arrow indicates the propagation direction, **K** of the incident light, whereas the blue arrow represents the oscillating vector of the electric component of the light, **E**. (**b–e**) The simulated E-field distribution in the monolayer C18-Au NP film versus different wavelength of the incident light. The color bar scales versus |**E**|^2^. The blue balls represent the Au NPs. **K**, the incident light direction, is pointing into the plane of the paper denoted by the yellow×.

In order to better assess the plasmonic properties of those Au NP films fabricated through the three-phase interfacial self-assembly technique, 3D finite-difference time-domain (FDTD) calculations were performed. As shown in Fig. 8(a), a plane wave of electric field, ***E***, polarized along the x-axis propagates along the vector, ***K***, perpendicular to the Au NP film, which lies in xy-plane (refer to Figure S5 for details of the model setup). Figure 8(b–e) shows the electric field (E-field) intensity (*|**E***^*2*^|) distribution obtained from the FDTD calculations of the C18-Au NP film. The gap between adjacent Au NPs was set at 2.6 nm, based on the particle center-to-center distance of the C18-Au NP film calculated from SAXS. It is clear from Fig. 8(c) that the maximum E-field intensity occurred at the incident light wavelength, *λ* = 652 nm, indicating that the C18-Au NP film is resonantly excited at 652 nm. At off-resonance wavelengths (e.g. *λ* = 450 nm, 850 nm and 1200 nm) the E-field intensity is much weaker, as seen from Fig. 8(b,d,e). This is in a good agreement with the UV-vis-NIR spectrum of the C18-Au NP film (Table 2), in which the SPR peak occurred at 659 nm. The similar FDTD simulation results were obtained for the other four Au NP films. Seen from Fig. 8(c), when in resonance with the 652 nm light, the most intensive E-field is distributed between adjacent Au NPs which are aligned at the angle of ±30° with the E-field. The gigantic enhancement of the E-field in the finite interparticle gaps can be attributed to the constructive interference of the induced dipole plasmon resonance of each neighboring particle^64^. Different for the particle dimer SERS substrate, in which only a single hot spot was shared by a pair of adjacent NPs, more than one hotspots coexisted on a constituent particle in the NP monolayer due to the periodically-aligned interparticle gap^65^,^66^. Thus, our FDTD simulation here shall provide new insight into the design of plasmonic platforms based on NP superlattice structures. The comparison between UV-vis-NIR absorbance from FDTD simulation (solid line) and from experiment (symbol) is shown in Fig. 7. The SPR maximum of the experimental spectra agrees well with the FDTD results (also refer to Table 2) The good agreement of experiment and 3D FDTD simulations indicates the SPR shift observed here is intrinsic to the material properties (e.g. ligand dielectric constant) and Au NP stack geometries. It is worth emphasizing that the slight discrepancy (<10%) between experiment and the FDTD simulation regarding the SPR maximum may come from deviation between simulated and actual properties such as particle uniformity and edge roughness.

These two factors may also contribute to the difference of the full width at half maximum (FWHM) between experiment and FDTD simulation for all five Au NP films (Table 2). The larger FWHM in experimental observation might be due to a broader interparticle space distribution of different Au NP films than that of the ideal Au NP HCP superlattice simulated by FDTD. Interestingly, the FWHMs of the SPR peaks of the OA-Au NP film and the PS-Au NP film are smaller than those of alkylamine-Au NP films, indicating narrower interparticle distance distributions (i.e. enhanced film ordering) for the former two Au NP films. This point can also be validated by the smaller standard deviation of the two films shown in Table 2. In our previous report, we found that the localized ordering of the Au NP films increased with the surface alkyl chain length (i.e. interparticle distance)^20^. And this phenomenon was attributed to the decreasing interparticle electrostatic repulsion with increasing interparticle space, where the electrostatic repulsion among Au NPs brought by the remaining negative Au NP surface charge was considered as a major factor of the disorder of the monolayer Au NP films^20^,^21^. However, the ordering of the OA-Au NP film, which has a smaller interparticle distance than that of alkylamine Au NP films, is better than the alkylamine Au NP films. This could be due to the more loosely packed OA better filling minor voids created by the polydispersity of the particles.

**Figure 9:**
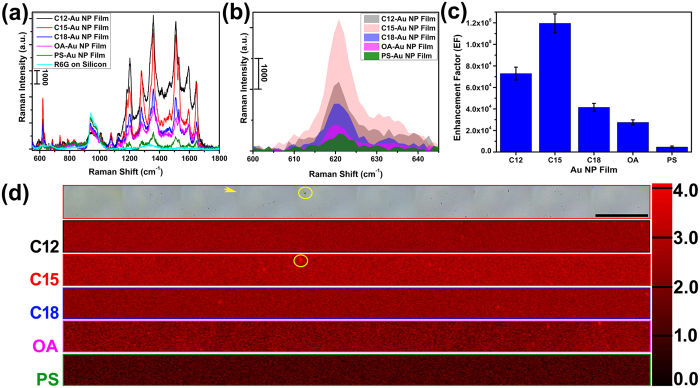
(**a**) SERS spectra of R6G on various substrates. (**b**) SERS spectra of the C-C-C in plane bending band (at ~622 cm^−1^) collected from different Au NP monolayers. (**c**) The calculated *EF* based on the integration of 622 cm^−1^peak of each Au NP film. The error bar represents the related standard deviation based on five measurements at different locations on the same sample. (**d**) Microscope image of the C15-Au NP film (top panel) and the Raman mapping of the aromatic C-C stretching band (at ~1359 cm^−1^) integration for different Au NP films on silicon wafer. The Raman mapping on C15-Au NP film was collected from the same region shown in the microscope image. The scale bar is 0.1 mm for all images. The color bar represents the integration of the Raman spectra at 1359 cm^−1^. The periodic vertical lines indicated by yellow arrow (top panel) is due to the artificial effect of the sample stage stepwise moving during the microscope images collection. The Raman mapping was not affected by this effect. The yellow circles call out local high concentration of R6G due to uneven distribution during drying.

It has been shown in many studies^67^,^68^ that when analyte molecules of interest are trapped in the locally enhanced electromagnetic (EM) field, their characteristic Raman spectrum are significantly enhanced. This surface enhanced Raman scattering (SERS) phenomenon is closely related to LSPR of the substrate, and has found vast applications in detecting low-concentration analytes in many fields such as energy storage^69^, cell labeling^70^, explosives/toxics detection^71^,^72^, catalysis^73^. Randomly deposited noble metal nanoparticle films are capable of enhancing Raman signal, yet hurdles exist to quantify the enhancement, largely due to the lack of information of the areal density and hot spot distribution^67^. The well-ordered hot spots across the monolayer Au NP films fabricated in this study, however, offer an ideal route for fabricating SERS substrates based on large-scale ordered Au NP arrays. R6G was used as a model Raman reporter in order to evaluate the SERS performance and the uniformity of the Au NP monolayers deposited on the silicon substrate. Shown in Fig. 9(a), all five Au NP monolayers exhibit remarkable Raman band intensity, whereas the same amount of R6G on silicon wafer does not show prominent peaks related to R6G, which indicates that the Au NP monolayers fabricated using air/water/oil system are promising SERS substrates. In contrast to the greatly enhanced R6G vibrational bands, no characteristic Raman spectra from the surface ligand was observed for each of the Au NP film (Figure S4).

To quantify the Raman enhancement of these films, the enhancement factor (EF) was calculated by^27^,^74^


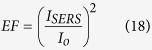


where *I*_*SERS*_ is the background-corrected SERS band integration at a certain Raman shift, and *I*_*o*_ is the normal Raman peak integration at the corresponding Raman shift. The peak representing C-C-C in-plane bend at around 622 cm^−1^ was chosen to calculate *EF* for each sample (Fig. 9(b)). As shown in Fig. 9(c), the *EF* of C15-Au NPs outperforms that of other films by a significant amount. The electromagnetic coupling between Au NPs in C15-Au NP monolayers is strongest due to its having the smallest particle-particle distance (refer to Table 1), which is consistent with our previous report^27^. This contributes to C15-Au NP monolayers having the largest *EF*, because the incident Raman laser wavelength (785 nm) is more enhanced by C15-Au NP monolayers with an SPR maximum of 683.6 ± 3.9 nm (Table 2) than any of the other monolayers. The *EFs* of C12-Au NP film and C18-Au NP film are 7.29 × 10^4^ and 4.15 × 10^4^, respectively. These values are close to ones obtained from their Au NP monolayer counterparts self-assembled at a water/oil interface^27^. For PS-Au NP film, due to its largest Au NP center-to-center distance and thus the reduced interparticle EM coupling, the calculated *EF* (4.65 × 10^3^) is an order magnitude smaller than that of the C12-Au NP film and C18-Au NP film. It is worth noting that however, the EF calculated for OA-Au NP film, which has a smaller interparticle distance than C12-Au NP film, is only 0.37 times that of the C12-Au NPs at 2.74 × 10^4^. This indicates that the SERS efficiency is not solely dependent on the interparticle space. In fact, multiple SERS mechanisms are commonly considered to contribute to Raman intensity amplification, namely the resonant enhancement of the incident beam, the resonant enhancement of the analyte signal, and the “chemical enhancement” due to analyte adsorption^75^. Although the EM-enhancement of incident light is considered as the largest contributor to the SERS intensity amplification, the chemical enhancement cannot be totally ruled out. Many studies have demonstrated that the SERS efficiency is closely related to non-EM factors, such as the adsorption of the analyte to the Au surface^76^,^77^, and the adsorbed analyte molecular orientation^78^. As mentioned earlier, the surface OA ligand in the Au NP monolayer has a different chain conformation than that of the alkylamine-Au NP film. The reduced SERS intensity for OA-Au NP film is possibly due to the reduced chemical enhancement (i.e. the adsorbate or orientation of the R6G on OA-Au surface less favors the SERS than on alkylamine-Au NP monolayers). The second possible contribution to the significant decrease of the EFs of the OA-Au NP film and that of the PS-Au NP is the interaction of the unsaturated carbon-carbon double bond of the OA/aromatic rings of the PS with the Au NP surface conduction electrons. It has been found that a donation of the π orbital electron density can occur to the half-filled s orbital due to the small d-s promotion energies of the gold s^1^d^10^ electron configuration^79^,^80^,^81^. This type of electronic interaction between the OA/PS and the Au NP may result in a weakened induced dipole momentum of the Au surface electron oscillation and consequently a reduced EM field. The findings abovementioned demonstrate the flexible EF tunability of the SERS-active substrates based on the Au NP monolayer in this study. In addition, it shall provide invaluable information on the screening of the ligand type in fabricating the NP-based SERS substrates in order to gain optima SERS performance.

In order to test the homogeneity of the Au NP monolayer SERS performance, line-scanning Raman mapping technique was used to probe a large area of the Au NP monolayers with R6G deposited on top. As shown in Fig. 9(d), an area of 1200 × 60 μm was scanned, and the Raman mapping was indexed to the integration of the aromatic C-C stretching band at 1359 cm^−1^ for each sample. The top panel of Fig. 9(d) shows a microscopic image of a randomly-selected region on C15-Au NPs. It is almost featureless, meaning that the Au NP film is evenly distributed and gives almost the identical diffraction of the white light across the film. The small dark dots observed occasionally (yellow circle) on the Au NP film is due to the uneven deposition of R6G during drying, which is manifested by the corresponding mapping file intensified region (yellow circle). The fairly even distribution of the 1359 cm^−1^ peak intensity integration can be seen from Fig. 9(d), demonstrating that the SERS performance of those Au NP monolayers is homogeneous across the mapped area. The relative mapping intensity variation for those films is consistent with their calculated EF. In this study, the dependence of the SPR maximum on the ligand dielectric constant, and interparticle space suggests that the collective SPR of these Au NP films can be flexibly manipulated by applying organic ligands of different chain lengths and dielectric constants, potentially benefiting the development of the nanoscale devices with predesigned optical properties ranging from near-field infrared imaging, plasmon enhanced absorption for photon harvesting, surface-enhanced infrared absorption spectroscopy^82^, and surface-enhanced Raman spectroscopy^83^. These results clearly show that the water/air/oil three-phase self-assembly technique developed here holds great potential in fabricating large-area monolayer Au NP films of controllable EF and promising homogeneity.

## Conclusions

In summary, a fast, efficient self-assembly method has been developed whereby aqueous nanoparticles form ordered Au NP monolayers of long-range ordering in an air/water/oil three-phase system. We proposed that the driving force for the formation of monolayer Au NP films on the water surface was the minimization of Helmholtz energy of the total Au NPs in the three-phase system. We expect this three-phase approach to be applicable to any material system that satisfies the free energy minimization condition, including Au NPs with alkylthiol ligands. The particle center-to-center distance could be flexibly manipulated by attaching amine ligands of different types and lengths to the Au NP surface. On the basis of interparticle spacing, the Au NP monolayers fabricated in this study also offer an ideal frame to study the NP surface ligand scaling behavior, which merits the fundamental understanding on the NP surface ligand conformation in the superlattice. The surface plasma resonance of the monolayer Au NP films could also be flexibly tuned accordingly. Due to the excellent long-range ordering, those Au NP monolayers hold great potential in functioning as EF-tunable and uniform SERS substrates. Our findings open up a new avenue in scaling up the manufacture of plasmonic nanoparticle devices with precisely designed optical properties. It should be emphasized that this method was developed based on the surface energy minimization mechanism, thus should be capable of universally applying to the self-assembly of other aqueous NPs of various shapes and sizes.

## Methods

### Materials

Gold (III) chloride trihydrate (HAuCl_4_·3H_2_O, ≥99.9% trace metals basis), sodium citrate dihydrate (HOC(COONa)(CH_2_COONa)_2_·2H_2_O, ≥99%), ethanol (ACS reagent, ≥99.5%), acetone (HPLC grade, ≥99.9%), toluene (anhydrous, ≥99.8%), n-hexane (anhydrous, 95%), chloroform (anhydrous, ≥99.5%), benzene (ACS spectrophotometric grade, ≥99%), dodecylamine (CH_3_(CH_2_)_11_NH_2_, ≥99%), pentadecylamine (CH_3_(CH_2_)_14_NH_2_, 96%), octadecylamine (CH_3_(CH_2_)_17_NH_2_, 97%), oleylamine (≥98%), Rhodamine 6G (R6G, dye content 99%) and amine terminated polystyrene (average Mn = 5000, PDI ≤ 1.2) were purchased from Sigma-Aldrich and used as received. Deionized (DI) water (18.2 MΩ·cm at 25 °C) was supplied by a Millipore water purification system. Gas-tight containers (10 × 10 × 5 cm^3^, Snapware) were purchased from McMaster. Silicon wafers (5 × 5 mm^2^, purchased from Ted Pella) were thoroughly cleaned with Piranha solution at 60 °C. (Caution: Piranha solution is highly corrosive and reacts violently with organic matter!) For all experiments, glassware was cleaned in a base bath (a mixture of 1 L DI water, 4 L ethanol and 250 g NaOH). Teflon coated magnetic stir bars (VWR) were cleaned with acetone. After cleaning, all components were rinsed with DI water and dried overnight at 100 °C before use.

### Au NP synthesis

Gold nanoparticles (6.35 ± 0.6 nm in radius) were synthesized using a modified “Turkevich” method reported elsewhere^28^,^84^. Briefly, aqueous gold chloride solution (200 mL, 0.5 mM) and sodium citrate solution (10 mL, 38.8 mM) were separately brought to boiling under vigorous stirring. Then the latter was quickly injected to the former. The mixture was kept boiling for 20 minutes until the colloidal color remained unchanged.

### Monolayer Au NP film formation

An organic solution of amine (10.4 μM, 4.5 mL) was added to a petri dish (*Φ* = 5.5 cm). Then, aqueous gold colloid (1 mL) was carefully injected to the bottom of the petri dish where it remained as a convex drop which protruded through the organic phase, i.e., it was exposed to air (as shown in Fig. 1(a)). Ethanol was added dropwise (0.5 mL) to the water/hexane interface at about 0.1 mL/min. Small Au NP islands of golden sheen appeared at the water/hexane interface (Fig. 1(b)). The islands rapidly moved from the water/oil interface to the water/air interface to form a larger Au NP film (Fig. 1(c)). This process is shown in the supporting video. The time needed for the formation of the large-scale Au NP monolayers in this air/water/oil system was less than 10 min. After drying the surrounding organic solvent, the film was transferred to solid substrates for more in depth studies by a “drain-to-deposit” method reported previously^20^.

### Au NP film characterization

The Au NP films were deposited on silicon wafers for scanning electron microscopy (SEM) and Raman spectroscopy; on glass slides for UV-vis-NIR spectroscopy; on carbon-coated copper grids (200 mesh) for transmission electron microscopy (TEM) and small angle X-ray scattering (SAXS). SEM images were taken on a JEOL 7401 SEM at an accelerating voltage of 20 kV and a probe current of 20 μA. Raman spectra were collected using a confocal Raman microscope (inVia Renishaw), equipped with a diode laser source (785 nm, 300 mW) and an automatic sample stage with submicron accuracy. The total laser power impinging on a sample surface was less than 2.5 mW, and the exposure time was 10 s. A line-laser mode was chosen for Raman mapping, with a mapping step 1.2 μm and a scanning range 1200 × 60 μm^2^ for each sample. A drop of freshly-made R6G/ethanol solution (1 μL, 100 μM) was drop cast on each substrate, and dried in ambient condition before each experiment. The R6G areal density on the substrate is estimated at 7 × 10^−13^ mol mm^−2^ based on the diameter of the R6G/ethanol droplet. The Raman spectra were analyzed using Renishaw WiRE 3.4 software. A UV-vis-NIR spectrophotometer (Agilent, Cary 5000) was used to analyze the monolayer Au NP films on glass slides. The glass slide was mounted on a sample holder perpendicular to the incident light beam (with a deuterium source and calcite polarizer accessory) during measurement. The aperture size was 1 mm. A JEOL 2011 was used to collect TEM micrographs at an acceleration voltage of 200 kV and a probe current of 113 μA. In order to determine the interparticle spacing, radial distributions of Au NPs in 2D TEM images were evaluated using ImageJ. At least 5 TEM images were analyzed for each amine-Au NP film. SAXS measurements were performed on a Nano STAR AXS system (Bruker) with 3-pinhole collimation. At least 3 SAXS samples were analyzed. All experiments were conducted at room temperature (25 °C) under vacuum (10^−2^ mbar) using Cu Kα radiation with wavelength, λ = 1.542 Å, generated at 45 kV and 650 μA. The sample-to-detector distance was 1045 mm. In addition to monolayers on carbon-coated copper grids, scattering data was also collected for aqueous Au NP colloid using the method documented elsewhere^28^.

### Finite-difference time domain (FDTD) simulation

Three dimensional (3D) FDTD simulations were performed with commercially available software, Lumerical FDTD Solutions, which is based on a numerical solution of the four Maxwell’s equations. Shown in Figure S5, the simulation objects were arrays of spheres with local HCP structure deposited on a transparent glass slide of refractive index 1.52. The geometric dimensions of the sphere arrays were based on the results of SAXS measurements. Based on our previous study^28^, the diameter of each sphere is 12.7 nm and the center-to-center distance is listed in Table S2. The dielectric parameters of gold were taken from the tabulated values by Johnson and Christy in the spectrum range from 450 nm to 1000 nm^85^. A comparison of the gold dielectric function between the tabulated value in ref. 85 and that simulated by FDTD was shown in Figure S6. The local refractive index of the sphere surface was set according to the values of the ligand (Table S2). In each case, the light source was a plane wave that propagated along the –z direction and was polarized along the x direction (Figure S5(a)). The wavelength range was between 450 nm and 1000 nm. Periodic boundary conditions (BC) were imposed along xz-plane and yz-plane of the simulation region, while a perfectly matched layer BC was set along xy-plane. The numerical mesh size was set to 0.7 nm for all cases, such that there were at least two mesh points between even the most narrow particle gaps. Refer to supporting information for details.

## Additional Information

**How to cite this article**: Yang, G. and Hallinan, D. T. Gold Nanoparticle Monolayers from Sequential Interfacial Ligand Exchange and Migration in a Three-Phase System. *Sci. Rep.* **6**, 35339; doi: 10.1038/srep35339 (2016).

## Supplementary Material

Supplementary Information

Supplementary Video S1

## Figures and Tables

**Table 1:** The center-to-center distance between adjacent Au NPs calculated from pair correlation function and that from 1D SAXS profiles.

Center-to-center Distance (nm)	C12-Au NP Film	C15-Au NP Film	C18-Au NP Film	OA-Au NP Film	PS-Au NP Film
Pair Correlation	14.4 ± 0.2	14.3 ± 0.2	15.2 ± 0.1	14.2 ± 0.2	16.7 ± 0.2
SAXS	14.7 ± 0.2	14.5 ± 0.3	15.3 ± 0.1	14.6 ± 0.2	17.0 ± 0.2

**Table 2:** Comparison of the SPR maximum and the full width at half maximum (FWHM) between the experimental results and the FDTD simulated results.

Sample ID	Experimental SPR Max (nm)	Experimental FWHM (nm)	FDTD SPR Max (nm)	FDTD FWHM (nm)
C12-Au NP Film	678.4 ± 3.9	359.0 ± 10.0	665.0 ± 0.4	112.9 ± 6.5
C15-Au NP Film	683.6 ± 3.9	342.0 ± 7.9	686.0 ± 0.3	162.4 ± 8.1
C18-Au NP Film	658.8 ± 1.2	327.0 ± 7.5	652.0 ± 0.1	147.4 ± 4.6
OA- Au NP Film	681.8 ± 1.8	194.0 ± 3.1	680.0 ± 0.2	110.3 ± 2.6
PS- Au NP Film	601.7 ± 2.1	149.7 ± 1.5	610.8 ± 0.9	126.1 ± 10.9
