# Supplementary material for: Gold Nanoparticle Monolayers from Sequential Interfacial Ligand Exchange and Migration in a Three-Phase System
Source: Sci Rep. 2016 Oct 20;6:35339. doi: 10.1038/srep35339 (PMC5071885; doi:10.1038/srep35339)
Supplement: Supplementary Information [file srep35339-s1.doc]

**Supporting information for “Gold Nanoparticle Monolayers from Sequential Interfacial Ligand Exchange and Migration in a Three-Phase System”**

Guang Yang, Daniel T. Hallinan Jr.*

Florida State University, Aero-Propulsion, Mechatronics & Energy Center, 2003 Levy Avenue, Tallahassee, FL 32310, USA; Florida A&M University - Florida State University College of Engineering, Department of Chemical and Biomedical Engineering, 2525 Potsdammer Street, Tallahassee, FL 32310, USA.

Electronic address: [dhallinan@fsu.edu](mailto:dhallinan@fsu.edu).

**The surface tension model derived from Pieranski**

The following content shows how we derive the Helmholtz free energy change for a nanoparticle that moves from the water phase to the water/air interface based on Pieranski’s method. ^10^

In a water/air system, the total free energy of a particle can be expressed as

$E_{relative}=\frac{E}{\pi R\gamma_{W/A}}=z_{relative}^{2}+2(a-b)z_{relative}+2a+2b-1$ , (S1)

where *R* is the radius of the particle, *γ_W/A_* is the surface tension at water/air interface, E is the absolute free energy value, *z_relative_* is the ratio of the particle center position versus water level over the particle radius, a is the ratio of the surface tension at particle/water over that at water/air interface (i.e. *a* = *γ_P/A_/ γ_W/A_*) and b is the ratio of the surface tension at particle/water interface (i.e. *b* = *γ_P/W_/ γ_W/A_*).

Since Equation S1 is a parabolic equation with a positive coefficient of the square term, we can get the minimum value of *E_relative_* by equating its first derivative to zero as

$E'_{relative}=2\cdot z_{relative}+2(a-b)=0$ (S2)

Thus, the *E_relative_* has a smallest value at *z_relative_* = *-(a-b)*, which is

(S3)

The Helmholtz free energy change when one moves a particle from water to the water/air interface can then be expressed as

(S4)

Here the *E_relative W_* is the surface energy of a particle in water, which is equal to 4b.

Substituting Equation S3 into S4 allows one to get

$\Delta E_{A/W}=-\frac{\pi R^{2}}{\gamma_{W/A}}[\gamma_{W/A}-(\gamma_{P/W}-\gamma_{P/A})]^{2}$ (S5)

which is the form we used in the main article.

**Calculation of the oil/water interfacial energy**

Table S1 Values of the oil/air (*γ_O/A_*) and oil/water (*γ_W/A_*) surface tensions and the components of the oil surface tension (*γ_O_^d^* and *γ_O_^p^*) ^a^

| **Organic Solvent** | *γ_O/A_* | *γ_O/W_* | *γ_O_^d^* | *γ_O_^p^* |
| --- | --- | --- | --- | --- |
| **Hexane** | 17.9 | 50.6 | 17.9 | 0.0 |
| **Chloroform** | 26.7 | 50.7 | 26.7 | 0.0 |
| **Toluene** | 29.1 | 35.9 | 27.8 | 1.3 |
| **Benzene** | 28.2 | 50.9 | 28.2 | 0.0 |

^a^ All values are in mJ∙m^-2^ at 25 °C. The surface tension components of water were taken at *γ_O_^d^* = 21.5, *γ_w_^p^* = 50.4. ^11^ The surface tension components of oil were taken from reference ^12^. *γ_O/A_* and *γ_O/W_*
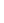
were calculated through Equation (8) and (9) in the main article, respectively. The water/air surface tension, *γ_W/A_* according to Equation (8), is the summation of *γ_w_^d^* and *γ_w_^p^*, which is 71.9.

**Au NP films formed using other organic solvents**


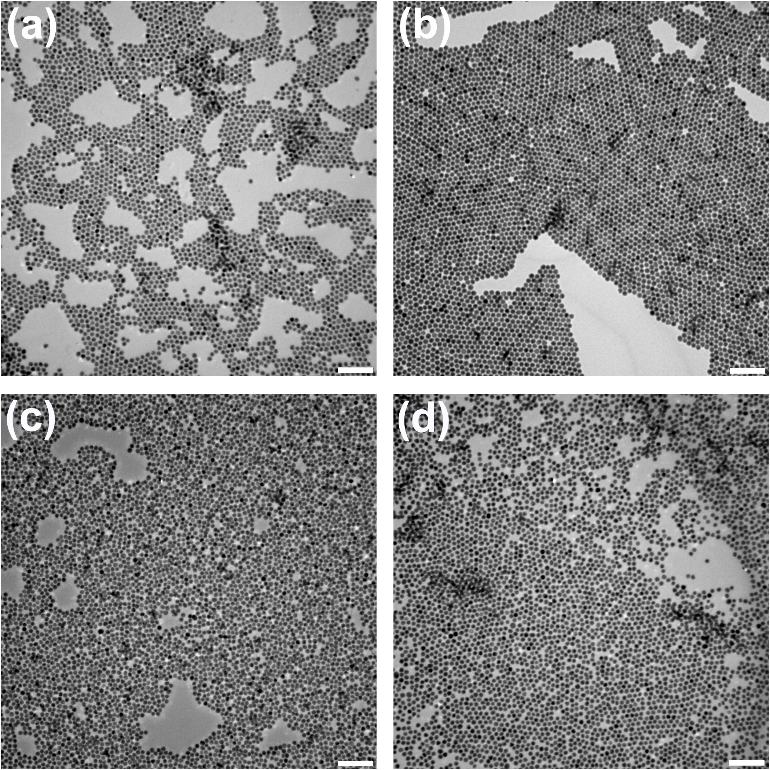


Figure S1. TEM image of the monolayer C18-Au NP film formed with (a) hexane, (b) chloroform, (c) toluene and (d) benzene as the oil solvent in an air/water/oil three-phase system. The scale bar is 100 nm for all images.

The TEM images in Figure S1 show that disordered Au NP films were formed if a single organic solvent was used. This is due to the mismatch of the interfacial energy of the oil phase with that of the water. More specifically, once the oil spread on the water surface, for those oils whose dispersion force is larger than that of the water (i.e. chloroform, toluene and benzene), the oil intermolecular attractive force is larger than that of oil/water intermolecular attraction. ^13^ The larger oil intermolecular attractive force results in the stacking of the adjacent oil molecules into discrete small oil droplets. Those small oil droplets floats on the water surface. Once trapped in the Au NP film, the oil droplets can create large void space in the film. For hexane, the dispersion force is 3.6 mJ∙m^-2^ lower than that of the water. Thus, the intermolecular attractive force among hexane molecules is smaller than that of the water. Once floating on water surface, hexane evaporates too fast due to the small intermolecular attraction. The instable evaporation of the hexane may cause irregular dissipative Au NP assemblies other than ordered monolayer Au NP film, as shown in Figure S1(a).

**The ligand concentration effect**

In this study, the ligand concentration in organic liquid was found to be an important parameter to affect the formation of the monolayer Au NP films at air/water interface, similar to what we have found in a previous report. ^14^

We first estimated the appropriate amount of ligands needed to cover the total Au NPs in 1mL aqueous colloid. We assume that all Au NPs have a hard spherical shape, with the average radius, r equal to 6.4 nm. ^14,15^ Also all Au^3+^ was reduced to form the Au^0^ (i.e. Au NPs), with negligible water loss during Au NP synthesis due to the reflux system. Based on mass balance, the number density of the aqueous Au NP colloid, ρ_NP_ is 4.53 × 10^12^ mL^-1^. ^15^ Also we assume that all Au NPs could be transferred to the oil/water interface, and only half of the Au NP surface was covered with ligand. ^14,16^ The surface area (*A_lig_*) occupied by a single ligand is estimated at 0.21 nm^2^. ^17^ Therefore, the ligand concentration in a 4.5 mL oil solution can be calculated by

$C_{lig}=\frac{1mL\cdot\rho_{NP}\cdot(4\pi r^{2}/2)}{4.5mL\cdot A_{lig}\cdot N_{A}}$ (S6)

with the Avogadro constant 6.02×10^23^ mol^-1^. The result of *C_lig_* is 2.1
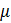
M. It has been found, however, that the Au NP films assembled at this theoretically optimistic ligand concentration have a large number of voids (Figure S1(a)), indicating that the amount of ligand attached to the Au NP surface was insufficient to balance the remaining electrostatic repulsive force. ^14^ The optimistic ligand concentration was 10.4
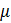
M, with which monolayer Au NP film of long range ordering can be formed (Figure S1(b)). The OA ligand concentration was also about 5 times as much as the theoretically optimistic ligand concentration.

**
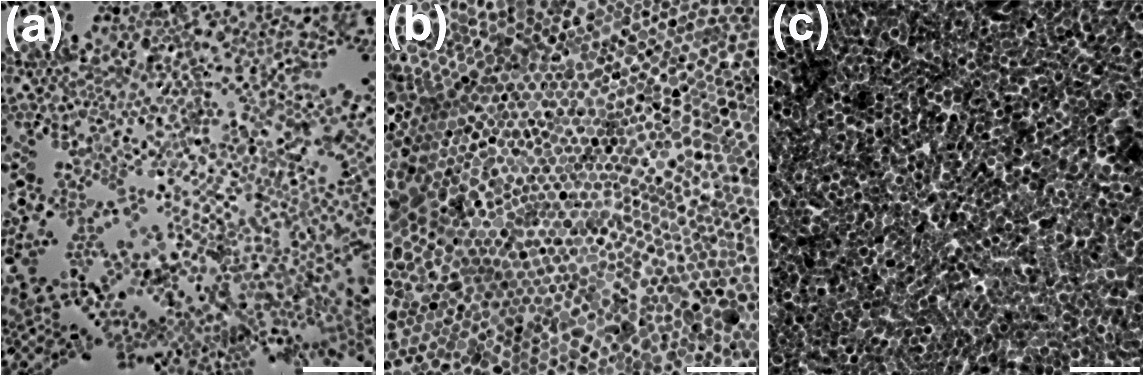
**

Figure S2. TEM images of the C18-Au NP film formed with ligand concentration of (a) 2.1
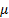
M, (b) 10.4
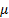
M and (c) 105
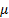
M in the oil phase. The scale bar is 100 nm for all images.

To further increase the ligand concentration to 105
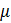
M, multilayer Au NP film was formed on the air/water interface (Figure S2(c)). Using the water/hexane interfacial self-assembly technique, multilayer Au NP films were formed with high ligand concentration due to the shift of the Au NP center from water/hexane interface to the hexane phase. A second NP layer could fill in the space leaving behind by the center-shifted NP, so that multilayer NP films could be formed. ^14,16^ The same high ligand concentration mechanism should apply to air/water/oil three-phase self-assembly system.

**The interparticle edge-to-edge distance**

**
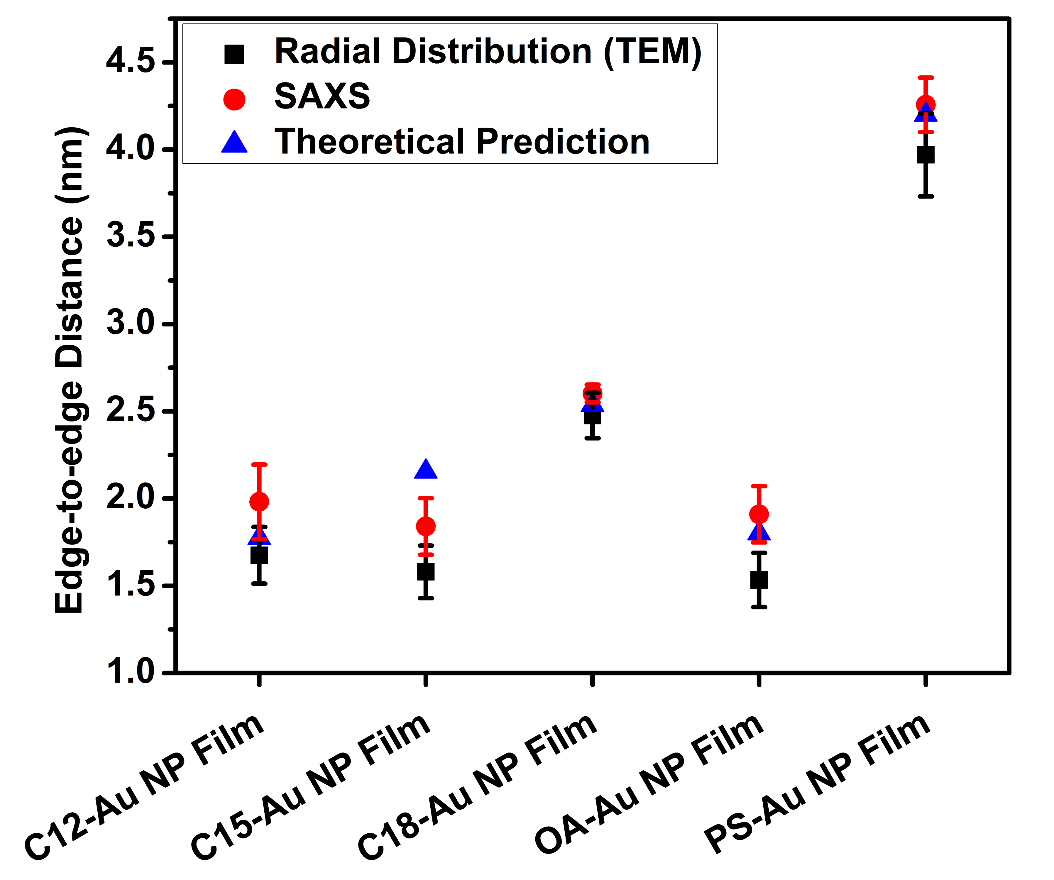
**

Figure S3. Plot of interparticle edge-to-edge distance of each Au NP film from the radial distribution, 1D SAXS profile, and theoretical prediction.

The interparticle edge-to-edge distance can be calculated using center-to-center distance by

$d_{e-e}=d_{c-c}-2r$ (S7)

with the average Au NP radius estimated at 6.35 nm. ^15^ The theoretical prediction of the interparticle edge-to-edge distance of alkylamine-Au NP film can be estimated the same as a single alkyl chain if we assume ligands on adjacent Au NPs have a fully interdigitated configuration.

It has been shown previously that the single alkyl chain of n carbon atoms can be estimated at ^18^

$L(nm)=0.127n+0.25$ (S8)

The theoretical prediction of the *d_e-e_* of OA-Au NP film is a single OA chain length estimated at 1.8 nm, and the *d_e-e_* of PS-Au NP film is the diameter of a single PS globule.

**PS contour length**

According to Rubinstein, the length of a fully extended polymer chain, *R_max_*, can be calculated by ^19^

$R_{\max}=n\cdot l\cdot\cos(\frac{\theta}{2})$ (S9)

where n is the number of the skeleton carbon bonds (see Equation (16) in the main article), l is the length of a single C-C bond (0.154 nm) and *θ* is the supplementary angle of the PS. The C-C bond angle for the atactic PS is between 112 and 120. ^20^

**Raman spectra of blank Au NP monolayers**


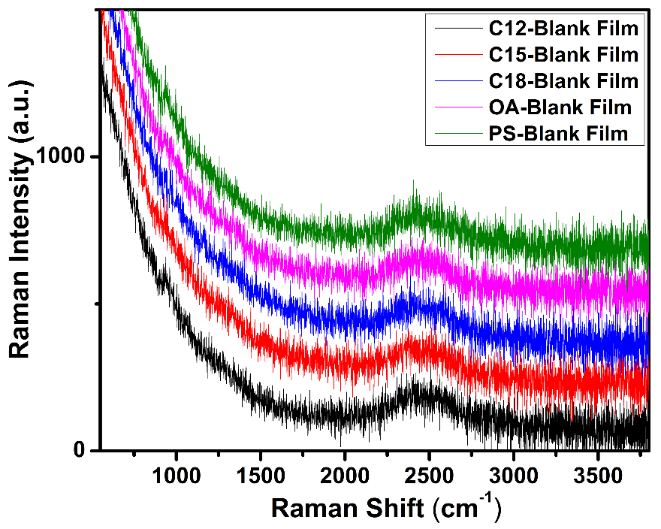


Figure S4. Raman spectra of various Au NP monolayer without R6G (plots are shifted for clarity).

Figure S4 shows the Rama spectra taken from the blank Au NP monolayers (i.e. no R6G was applied to the Au NP films). In sharp contrast to the Raman spectra of R6G enhanced by orders of magnitude on those films, no appreciable characteristic vibrational spectra from the amine ligands could be observed.

**FDTD simulation model setup**


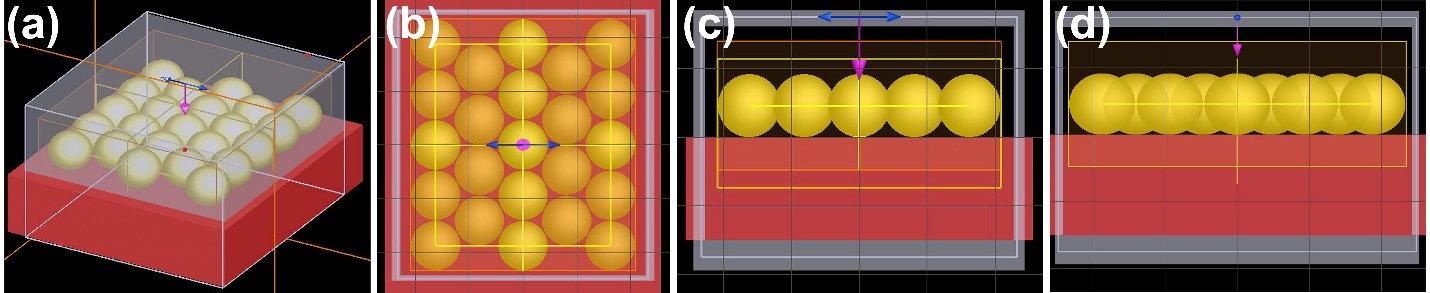


Figure S5. Schematic representation of the model setup in FDTD simulation. (a) 3D view of the simulation object in the Cartesian coordinate system. The red box stands for the glass slide. Golden spheres stand for the gold nanoparticles. The Au NP array is arranged in the xy plane, with z-axis perpendicular to the array pointing upwards. The grey square represents the simulated polarized light beam with wavelength ranging from 450 nm to 1000 nm. The pink arrow depicts the light wave propagation direction (-z direction) and the blue arrow indicates the electric field polarization in the x direction. (b) 2D view of the simulated object in the xy-plane. The yellow square represents the region where the FDTD simulation was carried out. (c) The xz-plane view and (d) the yz-plane view of the model.

Table S2. Particle center-to-center distance and refractive index used in FDTD simulation model setup for each Au NP film

| **Sample ID** | **Center-to-center Distance (nm)** | **Refractive Index** |
| --- | --- | --- |
| C12-Au NP Film | 14.7 | 1.437 ^a^ |
| C15-Au NP Film | 14.5 | 1.446 ^b^ |
| C18-Au NP Film | 15.3 | 1.449 ^b^ |
| OA-Au NP Film | 14.6 | 1.460 ^c^ |
| PS-Au NP Film | 17.0 | 1.590 ^d^ |

^a^from Reference ^1^, ^b^from Reference ^2^, ^c^from vendor (Sigma Aldrich) and ^d^from Reference ^3^.


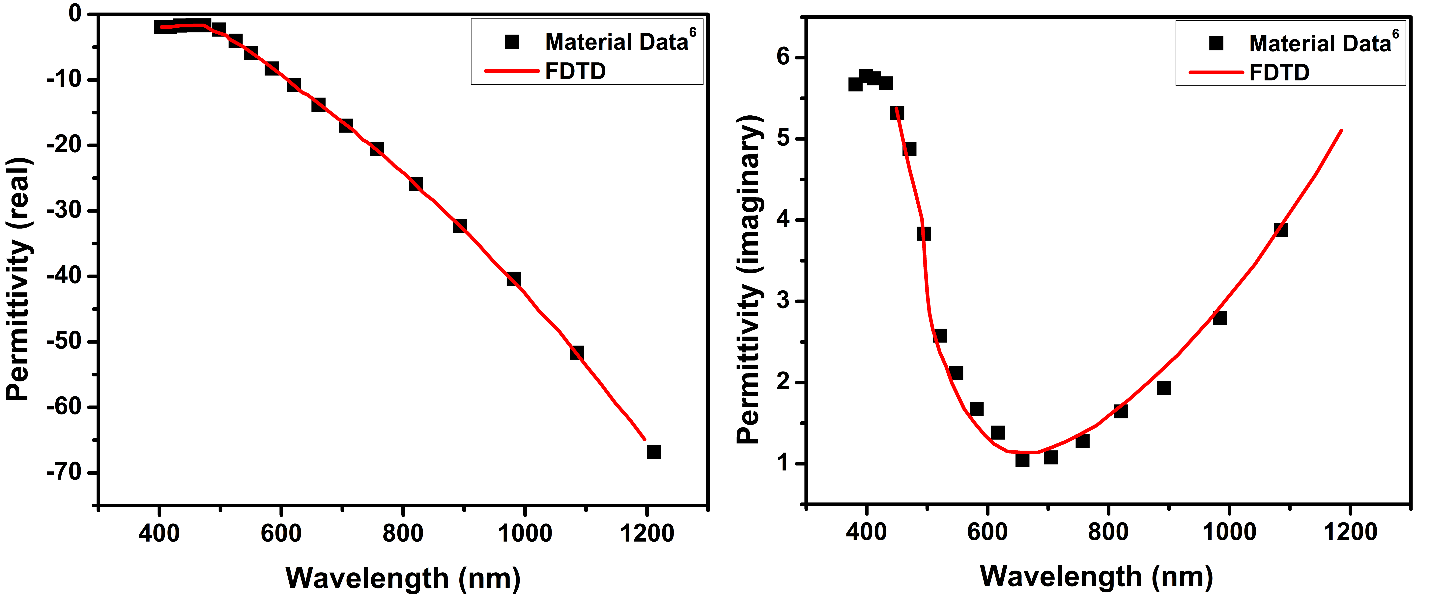


Figure S6. Comparison of the gold dielectric function versus wavelength between the tabulated value reported by Johnson and Christy ^4^ and that of FDTD simulation, with the left showing its real part and the right showing its imaginary part.

The Finite Difference Time Domain (FDTD) method, first developed by Kane S. Yee, ^5^ has become a state-of-the-art tool to solve Maxwell’s equations in complicated geometries. It calculates how the electromagnetic (EM) field propagates from the source through the physical structure of interest. The FDTD method employs finite differences as approximations to both spatial and temporal derivatives that appears in Ampere’s law and Faraday’s law. Thus, the EM fields and complex geometries that EM fields travel through are solved on a discrete mesh made up of so-called “Yee cells”. ^5^

In isotropic media, the differential forms of Maxwell’s equations can be written as ^6^

$\frac{\partial B}{\partial t}=-\nabla\times E$ (S10)

$\frac{\partial D}{\partial t}=-\nabla\times H-J$ (S11)

$D=\varepsilon E$ (S12)

$B=\mu H$ (S13)

where ***B*** is the magnetic flux density (Wb/m^2^), ***H*** is the magnetic field intensity (A/m), ***D*** is the electric flux density (C/m^2^) and ***E*** is the electric field intensity (V/m). The permittivity, $\varepsilon$ (F/m), the permeability, $\mu$ (H/m) and the current density, ***J*** (A/m^2^) are functions of space and time.

In Yee’s paper, ^5^ the problem was cast in two-dimension. For simplicity, the strategy of FDTD can be illustrated by a one-dimensional (1D) scenario. It is readily extended to 2D and 3D. ^7^

Consider a 1D space with only variations along x direction. Assuming that electric field only has a z component, so that the Faraday’s law and Ampere’s law can be respectively written as (based on Equation S10 and S11)

$\mu\frac{\partial H_{y}}{\partial t}=\frac{\partial E_{z}}{\partial x}$ (S14)

$\varepsilon\frac{\partial E_{y}}{\partial t}=\frac{\partial H_{y}}{\partial x}$ (S15)

Equation S14 and S15 indicate that the derivative of magnetic field with respect to time can be put as the spatial derivative of the electric field, and *vice versa*. Equation S14 is used to advance the magnetic field in time, and Equation S15 is for advancing the electric field. Yee used a so-called leap-frog method to advance these two fields (*i.e.* a variable is advanced and then the other, followed by repeating the process).

The first order derivatives in Equations S14 and S15 can be discretized in time and space to yield a finite difference form, which we will express as follows,

$H_{y}(x,t)=H_{y}(m\Delta x,q\Delta t)=H_{y}^{q}[m]$ (S16)

$E_{z}(x,t)=E_{z}(m\Delta x,q\Delta t)=E_{z}^{q}[m]$ (S17)

Here, $\Delta x$is the spatial increment, with the index *m* corresponding to the special step. Likewise, $\Delta t$is the temporal increment, with the index *n* corresponding to the temporal step.

The central-difference approximation of the first order derivatives of a function, *f(x) at x=x_o_* is given by,

$\frac{df(x)}{dx}|_{x=x_{o}}\approx\frac{f(x_{o}+\frac{\delta}{2})-f(x_{o}-\frac{\delta}{2})}{\delta}$ (S18)

if the step size $\delta$= *m*Δx is sufficiently small. The terms containing higher orders of $\delta$are neglected.

Combining Equation S14, S16 and S18 yields

$\mu\frac{H_{y}^{q+\frac{1}{2}}[m+\frac{1}{2}]-H_{y}^{q-\frac{1}{2}}[m+\frac{1}{2}]}{\Delta t}=\frac{E_{z}^{q}[m+1]-E_{z}^{q}[m]}{\Delta x}$ (S19)

Similarly, combining Equation S14, S17 and S18 yields

$\varepsilon\frac{E_{z}^{q+1}[m]-E_{z}^{q}[m]}{\Delta t}=\frac{H_{y}^{q+\frac{1}{2}}[m+\frac{1}{2}]-H_{y}^{q+\frac{1}{2}}[m-\frac{1}{2}]}{\Delta x}$ (S20)

The Equation S19 and S20 of finite difference form, are derived based on Faraday’s law and Ampere’s law, respectively. And they can be put in a more convenient form as

$H_{y}^{q+\frac{1}{2}}[m+\frac{1}{2}]=H_{y}^{q-\frac{1}{2}}[m+\frac{1}{2}]+\frac{\Delta t}{\mu\Delta x}(E_{z}^{q}[m+1]-E_{z}^{q}[m])$ (S21)

$E_{z}^{q+1}[m]=E_{z}^{q}[m]+\frac{\Delta t}{\varepsilon\Delta x}(H_{y}^{q+\frac{1}{2}}[m+\frac{1}{2}]-H_{y}^{q+\frac{1}{2}}[m-\frac{1}{2}])$ (S22)

Equation S21 and S22 are update equations to advance the field components, *H_y_* and *E_z_*, respectively.

It is manifest from the update equations S21 and S22 that, the future value of the field components (*H_y_* and *E_z_*) depend only on their previous values, and neighboring electric field (for *H_y_*) or magnetic field (for *E_z_*).

For materials with finite magnetic conductivity, *σ_m_*, a current term should be added to the Faraday’s law,

$-\sigma_{m}H-\mu\frac{\partial H}{\partial t}=\nabla\times E$ (S23)

Likewise, the Ampere’s law becomes

$\sigma E+\varepsilon\frac{\partial E}{\partial t}=-\nabla\times H$ (S24)

where *σ* is the electric conductivity. Assuming the variation only in x direction and only and *H_y_* and *E_z_* components exist, the suitable update equations of the two fields based on the discretization of Equation S23 and S24 can be written as

$H_{y}^{q+\frac{1}{2}}[m+\frac{1}{2}]=\frac{1-\frac{\sigma_{m}\Delta t}{2\mu}}{1+\frac{\sigma_{m}\Delta t}{2\mu}}H_{y}^{q-\frac{1}{2}}[m+\frac{1}{2}]+\frac{\frac{\Delta t}{\mu\Delta x}}{1+\frac{\sigma_{m}\Delta t}{2\mu}}(E_{z}^{q}[m+1]-E_{z}^{q}[m])$ (S25)

$E_{z}^{q+1}[m]=\frac{1-\frac{\sigma\Delta t}{2\varepsilon}}{1+\frac{\sigma\Delta t}{2\varepsilon}}E_{z}^{q}[m]+\frac{\frac{\Delta t}{\varepsilon\Delta x}}{1+\frac{\sigma\Delta t}{2\varepsilon}}(H_{y}^{q+\frac{1}{2}}[m+\frac{1}{2}]-H_{y}^{q+\frac{1}{2}}[m-\frac{1}{2}])$ (S26)

Since the plane wave normal to the monolayer Au NP film (along the z direction in Cartesian coordinates) was used as the light source in this simulation, and the Au NP film has a periodic structure on x and y direction, it is reasonable to use a periodic boundary condition (BC) in the x and y directions. To mathematically describe this type of BC, we consider the 3D case, with variations on x direction at a given y and z position (*e.g. nΔy* and *pΔz*).

Assuming N_x_ discretized nodes exist as unit simulation area along x axis. At a given time t, the periodic BC of z components of electric field, E_z_ at x low boundary can be expressed as

$E_{z}|_{t}^{0,m\Delta y,p\Delta z}=E_{z}|_{t}^{N_{x},m\Delta y,p\Delta z}$ (S27)

And the corresponding BC at x high boundary can be written as

$E_{z}|_{t}^{N_{x}+1,m\Delta y,p\Delta z}=E_{z}|_{t}^{1,m\Delta y,p\Delta z}$ (S28)

The same periodic BC can be applied to other components at various directions for different fields.

While the periodic BC was applied to the x and y axes, the so-called perfectly matched layer (PML) BC was implemented along z direction. ^8^ The PML BC essentially simulates open boundaries (or reflectionless BCs) and can adsorb any type of wave that travels through the boundaries. However, it should be noted that, since the media at the boundary along the z direction in our simulation was set as the vacuum, for the PML region, the related parameter should be the same as vacuum (i.e. *σ* = *σ_m_* = 0). Thus, the update equations of different field components at this BC was reduced to Maxwell’s equation in vacuum (Equation S10 to S13) ^8^. To mathematically describe this BC, we consider the 3D case, with variations in the z direction at a given x and y position (*e.g. mΔx* and *nΔy*).

Assuming N_z_ discretized nodes exist as unit simulation area along z axis. And, in order to circumvent confusion, it was assumed that the plane wave light source was set above the z low boundary and propagate along positive z direction. At a given time t, the PML BC of x components of electric field, E_x_ at z high boundary can be expressed as

$E_{x}|_{t-\Delta t}^{m\Delta x,n\Delta y,N_{z}}=E_{x}|_{t}^{m\Delta x,n\Delta y,N_{z}+1}$ (S29)

Since the light source was set above the z low boundary, the components of the two fields were simply set as zero at the z low boundary.

The Courant number, *S_c_* is defined as the ratio of the maximum distance the energy can travel, cΔt over the spatial offset, Δx. It plays an important role in determining the stability of FDTD simulation. The mesh size in this study meet both the Courant stability condition, as well as a simulation accuracy requirement. The Courant stability condition is ^9^

$S_{c}=\frac{c\cdot dt}{\delta}\leq1$ (S30)

where *c* is the speed of the light in vacuum (3×10^8^ m/s), dt is the temporal step and δ is the spatial step size. In our study, dt is smaller than 0.00152 fs. The calculated Courant number with a mesh size of 0.7 nm is smaller than 0.41. Thus the mesh size setting meets the Courant stability condition. Given that the average NP size in this study is 12.7 nm, the mesh size is about 5.5% of a NP, which is reasonably small to guarantee the simulation accuracy. Further decreasing the mesh size did not result in appreciable change of the FDTD-simulated SPR maximum for each case, but significantly increased the simulation time. Thus, the mesh size of 0.7 nm is appropriate in our study.

**Supplementary Video S1**

Supporting Information, Video 1 for “Gold Nanoparticle Monolayers from Sequential Interfacial Ligand Exchange and Migration in a Three-Phase System”.

This video shows how Au NP monolayer is assembled at a water/air/oil three-phase system.

**Supporting information reference**
